# Supplementary material for: Development of a computer-aided design software for the quantitative evaluation of aesthetic damage
Source: PLoS One. 2019 Dec 18;14(12):e0226322. doi: 10.1371/journal.pone.0226322 (PMC6919621; doi:10.1371/journal.pone.0226322)
Supplement: S1 Code site — 1. (ZIP) [file pone.0226322.s001.zip › [EESC_jr][Projeto]Relatorio_07_21/reports/templates_zip/gentelella-master/vendors/validator/index.html]

Total Form Validation


Github

Vertical orientation


Disable alerts

# Forms: validation, styling & semantics

Name

?

Name must be at least 2 words

Occupation

?

An optional field. This field is only validated when it has a value.  
*Minimum 5 chars for this example.*

(optional)

HTML5 Regex

?

This field uses HTML5 "pattern" attribute to be validated.  
*"**\d+**" - only digits are allowed*

email

Confirm email address

Number

?

Number must be between 10 and 100

Date

Password

?

Should be of length 6 OR 8 characters

Repeat password

Telephone

?

Notice that for a phone number user can input a '+' sign, a dash '-' or a space ' '

Drop down selection

-- none --
Option 1
Option 2
Option 3

?

Choose something or choose not. what shall it be?

url

Checkboxes
I agree

Multifield

?

This is a multifield, which let the user input a serial number or credit card number for example, and the trick is to validate the combined result, which is stored in a hidden field

File upload

message


There is a hidden "Required" form field below, notice it will not be validated due to its lack of visibility. The reason for this is that sometimes there is a section in a form that is not visible until some action is taken, but you do not want to change all those fields' "required" attributes on-the-fly, so that is why.

Submit
